# The thermoception task: a thermal imaging-based procedure for measuring awareness of changes in peripheral body temperature

**DOI:** 10.1152/jn.00014.2023

**Published:** 2022-08-02

**Authors:** Alisha Vabba, Maria Serena Panasiti, Marina Scattolin, Marco Spitaleri, Giuseppina Porciello, Salvatore Maria Aglioti

**Affiliations:** ^1^Sapienza University of Rome and CLN2S@Sapienza, Istituto Italiano di Tecnologia, Rome, Italy; ^2^IRCCS, Santa Lucia Foundation, Rome, Italy; ^3^Department of Psychology, Sapienza University of Rome, Rome, Italy

**Keywords:** interoception, thermosensation, body temperature

## Abstract

Although thermal body signals provide crucial information about the state of an organism and changes in body temperature may be a sign of affective states (e.g., stress, pain, sexual arousal), research on thermal awareness is limited. Here we developed a task measuring awareness of changes in peripheral body temperature (thermal interoception) and compared it to the classical heartbeat counting task (cardiac interoception). With an infrared light bulb we delivered stimuli of different temperature intensities to the right hand of 31 healthy participants. Thermal interoceptive accuracy, i.e., the difference between participants’ real and perceived change in hand temperature, showed good interindividual variability. We found that thermal interoception did not correlate with (and was generally higher than) cardiac interoception, suggesting that different interceptive channels provide separate contributions to awareness of bodily states. Moreover, the results hint at the great salience of thermal signals and the need for thermoregulation in day-to-day life. Finally, thermal interoceptive accuracy was associated with self-reported awareness of body temperature changes and with the ability to regulate distress by focusing on body sensations. Our task has the potential to significantly increase current knowledge about the role of interoception in cognition and behavior, particularly in social and emotional contexts.

**NEW & NOTEWORTHY** We developed a novel task measuring awareness of changes in peripheral body temperature (i.e., thermal interoception). To avoid tactile confounds present in existing thermoceptive tasks, we used an infrared light bulb to deliver stimuli of different temperature intensities to the hand of participants and asked them to judge the perceived change in their hand temperature. Performance in the task showed good interindividual variability, did not correlate with cardiac interoceptive tasks, and was associated with self-reported thermosensitivity.

## Graphical abstract

**Figure:**
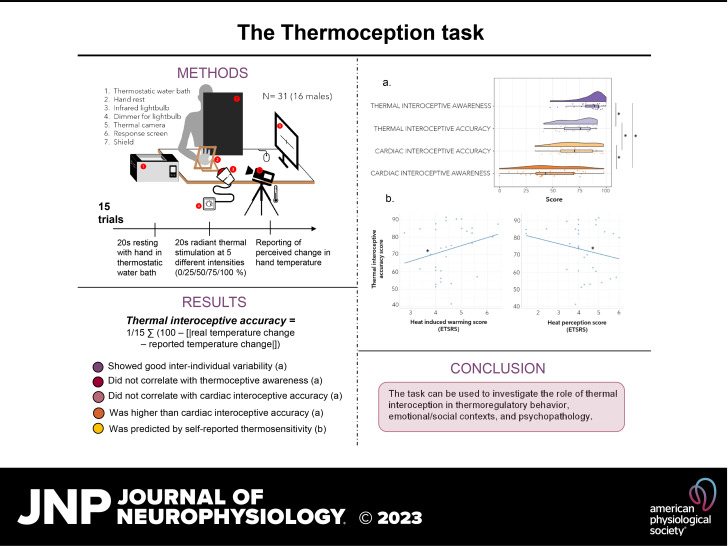

## INTRODUCTION

Recent years have shown increasing interest in the role that our awareness of body signals plays in emotion (for recent reviews see Refs. 1–3), psychopathology (for a review see Ref. 4), social cognition and behavior, such as empathy and perspective taking (5–9), social disposition (10), altruistic behavior (11), moral identity (12), and moral decision-making (13).

The term “interoception” is often used to describe the conscious and nonconscious processing of the physiological state of the body (14, 15), including preconscious effects of bodily states on cognition and the conscious awareness of these states. However, the precise the scope of this definition remains controversial. Whereas original, more restrictive, definitions refer exclusively to signals originating from the viscera, more recent, broader definitions encompass different types of information about the state of the body as a whole, which include skin surface signals like temperature, itch, pain, and sensual touch (see Ref. 16 for a critical review). Importantly, visceral and skin-mediated sensations present both similarities and differences: on the one hand, they activate a largely common neural network (e.g., the autonomic ganglia, medulla, thalamus, basal ganglia, and cortex) (17) and often share similar neural pathways (e.g., the spinothalamic tract; Ref. 15); on the other hand, they differ in many ways, such as whether they originate inside or outside the body, whether they derive from the autonomic, enteric, or somatic nervous systems, and which basal processing structures they use (i.e., loci in the nucleus of the solitary tract) (16). Problematically, research investigating interoception has focused almost exclusively on the cardiac domain, guided by the assumption that our awareness of signals from the heart can be used as a proxy for interoceptive ability in general. However, recent evidence suggests that the way we consciously process different bodily sensations (across the cardiac domain and other visceral and skin-mediated modalities) varies significantly. For example, studies have found that cardiac interoception does not correlate positively with respiratory interoception (18), gastric interoception (19, 20), pain (20, 21), or skin-mediated thermal interoception or sensual touch (21). Furthermore, tasks employed in the assessment of cardiac interoception and heartbeat estimation have recently come under severe controversy (22, 23), which raises questions about their psychometric validity (24–26).

The limitations of existing measures, and the notion that cardiac interoception cannot be generalized to all interoceptive domains, call for the need to develop new and noninvasive protocols tapping into different interoceptive channels. Here, we adopt the more inclusive definition of interoception as awareness of the physiological state of the entire body, with an appreciation of the importance of understanding how different physiological signals, from the heartbeat to skin-mediated sensations, influence our self-consciousness, cognition, and behavior. Within this scope, one aspect of interoception that has so far received little attention, regardless of its fundamental role in survival, is the perception of (peripheral and core) body temperature, a sensation that can be mediated by the external environment but can also arise from within the body (e.g., fever), and one that provides important information about the physiological state of the body.

Indeed, the survival of most species, humans included, depends on an optimal body temperature (27), and human beings make use of complex autonomic and behavioral means to survive and thrive while exposed to a wide range of environmental temperatures. In fact, dysregulations in body temperature may have serious, dramatic effects: for example, the mortality rate among people with compromised thermoregulation capacities, like the elderly (28), increases during extremely hot or cold seasons (29).

Recent evidence showed that visuo-thermal incongruencies reduced the illusory experience of owning a rubber hand and gave rise to visuo-thermal illusion effects, suggesting that thermal information is integrated with other bodily signals and crucially contributes to our sense of body ownership (30). Furthermore, our awareness of body temperature can affect our reliance on—and employment of—strategies aimed at regulating body temperature, i.e., behavioral thermoregulation (31, 32), and may be crucial to understanding disruptions of thermoregulation capacities that have been observed in a number of clinical conditions, including sleep disturbances (33–35), mood disorders (36–38), eating disorders (39, 40), and schizophrenia (41). Furthermore, awareness of temperature changes may be relevant in emotional and stressful contexts where they index changes in autonomic function. For example, increases in facial temperature have been associated with many affective states such as emotional arousal (42–44), anxiety (45), sexual arousal (46), stress (46, 47), and pain (46). In social scenarios, increases in facial temperature have been highlighted in response to social contact (48) and social ostracism (49, 50), during dishonest behavior (51, 52), and during in-group/outgroup categorization (53). Thus, tasks aimed at quantifying thermal interoception can be an important instrument in the study of thermoregulation and body awareness, with relevant applications for exploring the role of bodily changes in social and emotional contexts. To our knowledge, only few measures of awareness of temperature changes are available (21, 54). In virtually all of them, tactile thermal stimuli are delivered to the hand/forearm of participants, whose task consists of judging the temperature of an external object (such as a thermode) rather than that of their own body.

Here we developed a task to measure awareness of changes in peripheral body temperature without requiring the tactile modality or the estimation of the temperature of an external object. We used an infrared light bulb to deliver radial thermal stimulations of different temperature intensities, with the aim of inducing thermal changes to the palm of participants’ right hand. Subsequently, we asked participants to judge the extent of temperature change they perceived on their palm after each stimulation. We created indexes of *thermal interoceptive accuracy* (the capacity to accurately detect changes of hand temperature) and *thermal interoceptive awareness* (i.e., the correspondence between accuracy and confidence in task performance). Considering the salience of thermal signals in day-to-day life, where we are constantly exposed to environmental stimuli of different temperature intensities, we hypothesized that participants’ capacity in assessing thermal signals may be greater compared with the capacity to perceive cardiac signals, which are instead transient and faint and do not typically attract our attention unless something is wrong (e.g., tachycardia) (55). For this reason, we additionally collected a measure of *cardiac interoceptive accuracy* [through a heartbeat counting task (HCT)] and compared participants’ performance in the two tasks. We also collected self-report measures of interoceptive sensibility. We predicted a dissociation between thermal interoceptive accuracy and awareness and general interoceptive sensibility, mirroring the multidimensionality of cardiac interoception (56). To validate the task, we tested whether performance in the thermoception task could be predicted by questionnaires assessing *thermosensitivity*.

## METHODS

### Participants

A power analysis [power = 0.8; α = 0.05; effect size decided on the basis on the average correlations (*r* = 0.49) found by previous studies comparing tasks across different interoceptive modalities (19, 57, 58) using the *pwr* package for R] was used to estimate the sample size need for running a correlation analysis between the performance in the thermoception task and performance in a task of cardiac interoception (the heartbeat counting task; Ref. 59). According to the power analysis, a sample of 30 participants would be sufficient to detect a significant correlation between the tasks. A total of 47 participants took part in the study. However, the experimental protocol required strict control of the environmental temperature, and, because of Covid-19 regulations related to ventilation of the testing room, these conditions were violated in a great number of cases, leading to the elimination of the responses of 16 participants. The final sample consisted of 31 healthy volunteers (16 males; mean age = 27.21 yr, standard deviation = 4.71). The environmental temperature of the testing room was continuously checked by means of an external thermometer, and we managed to keep it constant throughout the experiment. The study was approved by the Ethics Committee of the Santa Lucia Hospital I.R.C.C.S. and was in accordance with the 1964 Declaration of Helsinki.

### General Procedure

Upon arrival at the laboratory, participants were informed that they were taking part in a study aimed at validating a task for the assessment of awareness of changes in body temperature. During the first 30 min, we asked participants to sit comfortably on a chair while they acclimatized to the environment and to the room temperature. During this time, they were asked to read and sign the informed consent form. They then performed the thermoception task and the heartbeat counting task, in counterbalanced order (see below for a detailed description of the 2 tasks). A series of self-reported measures were completed by the participants at home after participation, using the Survey Monkey platform (see below for a detailed description of the selected questionnaires).

### Behavioral Measurements

#### The thermoception task.

**Figure 1.**
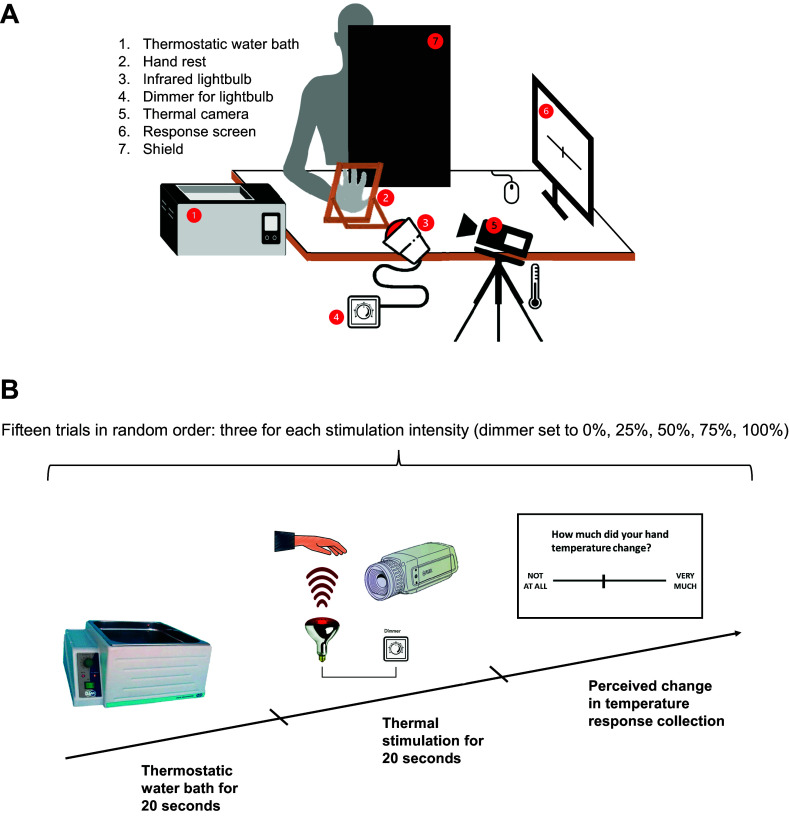
Experimental setup and design*. A*: the thermoception task setup. Participants are seated at a desk with a rest for their right hand. On their right is a thermostatic water bath, and on their left are a screen and a mouse for instructions and response collection. An infrared light bulb is attached to the desk at a 15-cm distance from the hand. A shield covers the light bulb from view, to avoid response bias due to visual cues. A thermal camera is used to record real changes in hand temperature during the task. *B*: illustration of a typical trial of the thermoception task. The task consists of 15 trials, 3 for each temperature intensity (0%, 25%, 50%, 75%, 100%). In each trial *1*) the participants insert their right hand for 20 s in the thermostatic water bath set at 31.5°C; *2*) participants dry their hand, place it on the stand, and receive the 20-s thermal stimulation, delimited by 2 acoustic tones; and *3*) participants report the perceived change in temperature from before to after the stimulation on a visuo-analog scale (VAS) ranging from 0 = “Not at all” to 100 = “Very much.”

Figure 1*A* depicts the task setup. Participants were seated at a desk with a hand rest for their right hand, which was secured to the desk and covered in thermal foil. Opposite the hand rest, at 15 cm, was an infrared light bulb (radiant heating; maximum power: 250 W, voltage: 240 V; dimmable; dimensions: 12 × 12 × 17.5 cm; weight: 160 g), which was connected to a dimmer that could be used to set the light bulb power to 0%, 25%, 50%, 75%, and 100%. To avoid responses being biased by visual cues, a shield was placed in front of the participant so that the light bulb and dimmer were covered from view. A FLIR A655sc thermal camera (FLIR Systems, AB, Sweden) was positioned next to the light bulb and recorded the right-hand temperature through each trial. On the participants’ right there was a thermostatic water bath (FALC Instruments model WB-MD15) set at a temperature of 31.5°C. Participants used the computer screen and mouse on their left side to rate how much their perceived hand temperature had changed after stimulation.

The experiment (Fig. 1*B*) consisted of 15 trials (3 per intensity of stimulation presented in randomized order), in which participants received thermal stimulations to the palm of their right hand. Before this, participants completed three practice trials: these served as a way for participants to familiarize themselves with the task procedure and with the highest stimulation intensity. As such, practice trials were not considered in the statistical analysis.

Each experimental trial was structured in the following three steps:

1)  At the beginning of each trial, for 20 s, participants immersed their right hand in the thermostatic water bath. Water temperature was set to 31.5°C to bring the hand temperature to a comparable and controlled baseline value before stimulation. During these 20 s, and in accordance with the randomized order generated by E-Prime, the experimenter set the dimmer, which was hidden from the participants’ sight, to the correct intensity.
2)  When prompted by the experimenter, the participants quickly and thoroughly wiped their hand with a paper towel and then placed it on the hand rest. Then, they were instructed to close their eyes and concentrate on the current temperature of their hand. In fact, participants’ task was to compare the temperature of their hand at two time points, which were signaled by two acoustic tones. Once participants were ready, the experimenter launched the first tone. After 2 s the light bulb was turned on and switched off after 20 s, i.e., when a second acoustic tone was delivered.
3)  After the second tone, participants were instructed to open their eyes and read the question “How much did the temperature of your hand change from the first to the second acoustic tone?” on the screen in front of them. Responses were provided with a left mouse click, along a visuo-analog scale (VAS) (*perceived temperature change VAS*), which ranged from 0 = “Not at all” to 100 = “Very much.”

At the end of the thermoception task, participants rated how accurate they believed their performance in the whole task to be. They did so on a second visuo-analog scale (*confidence rating VAS*), which in this case ranged from 0 = “Not accurate at all” to 100 = “Completely accurate.” Instructions, trial order, thermal camera triggering, and response collection were handled through E-Prime 2 software (Psychology Software Tools, Pittsburgh, PA).

The real change in participants’ hand temperature was recorded via a thermal camera using the software FLIR Research IR Max 4. The camera was triggered to start and stop recording respectively when the first and second acoustic tones were delivered, thus producing 15 files per participant.

#### The heartbeat counting task.

During the heartbeat counting task (HCT) (59), participants are asked to mentally count the number of heartbeats they perceive in the absence of physical cues (e.g., taking their pulse) during four intervals of different durations (i.e., 25, 35, 45, and 100 s). The order of intervals is randomized, and each is delimited by two auditory tones that the participants hear through a headset. At the end of each trial, participants enter the number of heartbeats they have felt with a computer keyboard. In the present study, participants were explicitly asked not to estimate the number of heartbeats they believed occurred during the trial but to only consider heartbeats they truly perceived. At the end of the experiment, participants rated their perceived level of accuracy in the task, using a visuo-analog scale (VAS) ranging from 0 = “Not accurate at all” to 100 = “Completely accurate.” Cardiac signals were recorded throughout the task with a physiological recording system (ADInstruments PowerLab and BIOPAC). Instructions, trial order, and response collection were handled by E-Prime 2 software (Psychology Software Tools).

### Self-Report Measures

The Experienced Temperature Sensitivity and Regulation Scale (ETSRS) (60) is a questionnaire designed to assess perceived thermosensitivity and autonomic or behavioral thermoregulatory activity. We selected and administered two subscales: the Heat-Induced Warming subscale is composed of five items and measures participants’ subjective perception of how quickly or intensely different body parts, like, for example, hands, feet, and torso, become warm when exposed to warm environments (e.g., “Compared to others, a warm environment gives me warm feet”), and the seven-item Heat Perception subscale measures the self-reported tendency to feel warm in different environments and/or situations, e.g., indoors, outdoors, in bed, when concentrating, when watching TV or reading (e.g., “Compared to others, I experience heat at home”). For both subscales, participants judge how much each sentence is true for them compared to other people, using a 6-point Likert scale ranging from 1 = “Much less” to 7 = “Much more.”

The Social Thermoregulation and Risk Avoidance Questionnaire (STRAQ-1) (61) is a survey developed to measure the importance of thermal cues and thermoregulatory biological drives in the formation of attachment and social relations (i.e., seeking physical contact and closeness to other people). We selected and administered three subscales: the High Temperature Sensitivity seven-item subscale, which measures how sensitive to—and bothered by—high temperatures participants are (e.g., “I can’t focus when it is too hot”); the Solitary Thermoregulation eight-item subscale, which estimates the tendency to use behavioral thermoregulatory actions to maintain thermoneutrality (e.g., “When it is cold, I wear more clothing than others”); and the Social Thermoregulation five-item subscale, which reflects the tendency to be physically close to—and to physically warm up with—other people (e.g., “I like to warm up my hands and feet by touching someone I am close to”).

The Multidimensional Assessment of Interoceptive Awareness (MAIA-2) (62) is a 37-item survey measuring interoceptive sensitivity, and it is composed of eight subscales, specifically, Noticing (4 items, e.g., “When I am tense, I notice where the tension is located in my body”), which measures awareness of uncomfortable, comfortable, and neutral body sensations; Not-Distracting (6 items, e.g., “I distract myself from sensations of discomfort”), assessing the tendency not to ignore or distract oneself from sensations of pain or discomfort; Not-Worrying (5 items, e.g., “I start to worry that something is wrong if I feel any discomfort”), measuring the tendency not to worry or experience emotional distress with sensations of pain or discomfort; Attention Regulation (7 items, e.g., “I can pay attention to my breath without being distracted by things happening around me”), which assesses the ability to sustain and control attention to body sensations; Emotional Awareness (5 items, e.g., “I notice how my body changes when I am angry”), measuring awareness of the connection between body sensations and emotional items; Self-Regulation (4 items, e.g., “When I feel overwhelmed, I can find a calm place inside”), measuring the ability to regulate distress by attention to body sensations; Body Listening (3 items, e.g., “I listen for information from my body about my emotional state”), assessing active listening to the body for insight; and Trusting (3 items, e.g., “I am at home in my body”), measuring the experience of one’s body as safe and trustworthy. Responses are collected on a 6-point Likert scale ranging from 0 = “Never” to 5 = “Always.”

The Body Perception Questionnaire—Short Form (BPQ-SF) (63) is a 22-item questionnaire assessing self-reported body awareness and autonomic reactivity. It measures the tendency to be aware of different body sensations and processes, such as bloating, stomach sensations, sweating, tremor, facial temperature (e.g., “My heart often beats irregularly,” “I feel shortness of breath”). Participants report how often they are aware of specific sensations using a 5-point Likert scale ranging from 1 = “Never” to 5 = “Always.”

### Analytic Plan

#### Measuring real and perceived hand temperature changes.

**Figure 2.**
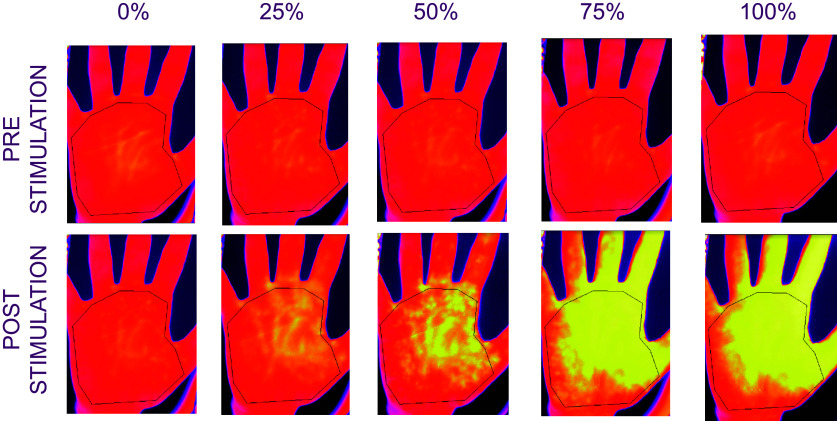
Thermal images of a representative participant’s hand. Thermal images were recorded via the thermal camera FLIR A655sc and FLIR Research IR Max software. The images were taken at baseline (2 s before stimulation) and at the end of the 20-s stimulation for each of the 5 intensity conditions (0%, 25%, 50%, 75%, 100%).

For every participant, each of the 15 thermal video files recorded by the thermal camera was visually inspected to make sure that participants were positioned correctly and did not move during the trial. An area of interest (AOI) was designed to comprise the palm of the participant’s hand with the software FLIR Research IR Max 4, using the base points of the palm and of each finger as reference points (see Fig. 2 for the AOI and a photographic example of thermal recordings across all stimulation intensities). Being directly affected by the stimulation, the defined area may be optimal for detecting thermal changes.

For each point in time, the average temperature (in degrees Celsius) within the area of interest was outputted into a single csv file. The first and last entries in the csv file were used respectively as a measure of hand temperature before and at the end of the thermal stimulation. We then calculated the difference in temperature between the two time points, i.e., the *real hand temperature change* for each trial.

The *perceived hand temperature change* for each trial was the score in the perceived temperature change VAS.

#### Effect of stimulation intensity on real and perceived hand temperature change and thermal interoceptive accuracy.

To test whether different stimulation intensities induced different real and perceived hand temperature changes and different scores in thermal interoception accuracy (see *Measuring thermal interoceptive accuracy and awareness*), we performed multilevel mixed-model analyses using the R package lme4 v 1.1–23. Multilevel mixed-model analysis was used to account for missing data due to technical issues with some of the trials. We constructed three separate models with the dependent variable respectively set to *1*) real hand temperature change, *2*) perceived hand temperature change, and *3*) thermal interoceptive accuracy, with stimulation intensity (0%, 25%, 50%, 75%, 100%) as the main within-subject predictor. Each trial of each participant was considered as a separate observation; therefore we entered 15 observations per participant. To deal with the nonindependence of the data set, the random intercept was set on Participant. We conducted a type III Wald ANOVA using the car package for R (R Studio version 4.0.2). Post hoc comparisons were performed with the R emmeans package and with Bonferroni correction for multiple comparisons.

#### Measuring thermal interoceptive accuracy and awareness.

We first transformed the real hand temperature change for each trial into a value between 0 and 100 (*standardized hand temperature change*), to better compare it with the score of perceived hand temperature change, which was rated on a 0–100 VAS. For each participant, we set the larger temperature change across all 15 trials to be the upper end of the range, while 0 indicated no change in hand temperature. We then transformed the real temperature change in each remaining trial according to the following formula:

$$\begin{array}{l} \text{standardized hand temperature change}\;\left(\text{SHTC}\right)=\left[\left|\text{real hand temperature change in trial}\right|\times 100\right]/\text{highest realhand temperature change}\\ \;\text{across all trials} \end{array}$$

SHTC values of 0 and 100 indicate, respectively, no change and the largest possible temperature change.

To have a unique measure of thermal interoceptive accuracy for each participant, we used the following formula:

$$\begin{array}{l} \text{thermal interoceptive accuracy}=1/N\;\text{of}\;\text{trials}\sum (100-[|\text{standardized hand temperature change in trial}-\text{perceived temperature change in trial}|]) \end{array}$$

Values of 0 and 100 indicate worst and best thermal interoceptive accuracy, respectively.

Thermal interoceptive awareness was then calculated with the following formula:

$$\begin{array}{l} \text{thermal interoceptive awareness}=100-[|\text{final confidence rating VAS}-\text{thermal interoceptive accuracy}|] \end{array}$$

Values of 0 and 100 indicate, respectively, no awareness and perfect awareness.

#### Measuring cardiac interoceptive accuracy and awareness.

Cardiac interoceptive accuracy was calculated as the difference of perceived to real heartbeats averaged across all trials, with the following formula:

$$\begin{array}{l} \text{cardiac interoceptive accuracy}=1/4\sum \;(100-\left[\left|\text{recorded heartbeats}-\text{perceived heartbeats}\right|\right]/\text{recorded heartbeats}) \end{array}$$

Scores closer to 100 indicate higher accuracy.

*Cardiac interoceptive awareness* was calculated as the difference between confidence judgments (scores in the final confidence rating VAS) measured on the overall performance in the HCT and the cardiac interoceptive accuracy score.

$$\begin{array}{l} \text{cardiac interoceptive awareness}=100-\;[|\text{final confidence rating VAS}-\text{cardiac interoceptive accuracy}|] \end{array}$$

Values of 0 and 100 indicate, respectively, no awareness and perfect awareness.

#### Comparison and correlation analysis between thermal and cardiac interoception.

To test the difference between thermal and cardiac interoceptive accuracy and awareness, we performed *t* tests. To test covariation between thermal interoceptive accuracy and awareness and cardiac interoceptive accuracy and awareness, we performed Bonferroni-corrected Pearson’s correlations and, where required, we calculated effect sizes and Bayes factor, using JASP software (JASP v.0.14.1). Bonferroni cutoff for significant correlations was determined by dividing the conventional *P* = 0.05 cutoff by the total number of correlations we computed (i.e., 4), which thus corresponded to *P* = 0.012. In all cases, as thermal interoceptive accuracy did not vary across active stimulations (25%, 50%, 75%, and 100%), which all differed from the baseline stimulation (0%), thermal interoceptive accuracy and awareness were calculated based on the average of the 13 active stimulation intensity trials (25%, 50%, 75%, 100%), excluding the baseline trials (0%).

#### Exploratory regression analyses between thermal interoception and subjective measures of thermosensitivity and interoceptive sensibility.

We performed linear regression analyses (linear models, LM) using the R package lme4 v 1-1–23 to investigate whether thermal interoceptive accuracy was predicted by subjective thermosensitivity and general interoceptive sensibility. Considering that thermal interoceptive accuracy did not vary across active stimulations (25%, 50%, 75%, and 100%), which all differed from the baseline stimulation (0%), we only included active stimulations in the models. Both models had thermal interoceptive accuracy (for active stimulations) as the dependent variable. The model assessing the relation between thermal interoceptive accuracy and thermal sensitivity corresponded to the following:

$$\begin{array}{l} \text{thermal interoceptive accuracy}∼\text{High Temperature Sensitivity}\;\left(\text{STRAQ-1}\right)+\text{Solitary}\;\text{Thermoregulation }\left(\text{STRAQ-1}\right)+\text{Social Thermoregulation }\left(\text{STRAQ-1}\right)+\text{Heat Perception }\left(\text{ETSRS}\right)\\ +\text{Heat-Induced Warming }\left(\text{ETSRS}\right) \end{array}$$

The model for investigating the role of interoceptive sensibility (MAIA-2 subscales and the BPQ-Short Form) in thermal accuracy was coded as follows:

$$\begin{array}{l} \text{thermal interoceptive accuracy}∼\text{Noticing}+\mathrm{Not}-\text{Distracting}+\mathrm{Not}-\text{Worrying}+\text{Attention Regulation}+\text{Emotional Awareness}+\mathrm{Self}-\text{Regulation}+\text{Listening}+\text{Trusting}+\mathrm{BPQ}-\text{SF} \end{array}$$

We conducted type III Wald ANOVAs using the car package for R.

## RESULTS

### Environmental Temperature

Average room temperature at the beginning of the experiment was 25.69°C (standard deviation = 1.17). The change in room temperature from the beginning to the end of the experiment was 0.645°C on average (standard deviation = 0.65). The average temperature of the thermostatic water bath was 31.53°C (standard deviation = 0.09) at the beginning of the experiment and remained constant throughout the experiment for all participants.

### Effect of Stimulation Intensity on Real and Perceived Change in Hand Temperature and on Thermal Interoceptive Accuracy

#### Real hand temperature change.

**Figure 3.**
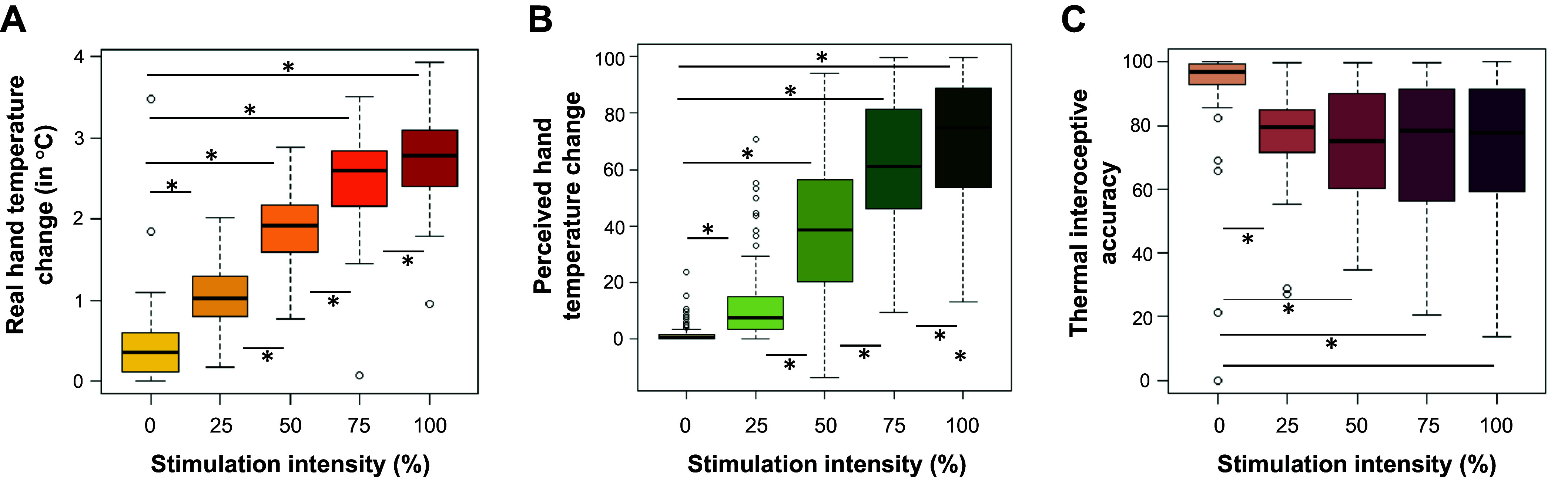
Box plots displaying differences in real and perceived change in hand temperature and interoceptive accuracy based on stimulation intensity. Values for each stimulation intensity are reported (0%, 25%, 50%, 75% and 100%). *A* represents differences in real change in hand temperature from before to after the stimulation, recorded by a thermal camera in °C. *B* shows differences in average perceived hand temperature [average of visuo-analog scale (VAS) scores]. *C* shows differences in thermal interoceptive accuracy. *P < 0.001.

Mean baseline hand temperature (i.e., taken at the first acoustic tone corresponding to the first real thermal frame) for all participants and stimulation types was 32.39°C (standard deviation =1.24). There was a significant main effect of stimulation intensity on the real hand temperature change (χ^2^ = 4,027.60, *P* < 0.001). Post hoc analysis revealed that the temperature change was significantly higher for all stimulation intensities (25%, 50%, 75%, and 100%) compared to the baseline, 0% stimulation intensity (smallest *b* = −0.65, smallest *t* = −14.72, all *P* values < 0.001). Furthermore, real hand temperature change was significantly higher for the 50% compared to the 25% stimulation intensity (*b* = −0.841, *t* = −18.99, *P* < 0.001), for the 75% compared to the 50% stimulation intensity (*b* = −0.62, *t* = −14.01, *P* < 0.001), and for the 100% compared to the 75% stimulation intensity (*b* = −0.24, *t* = −5.48, *P* < 0.001) (Fig. 3*A*).

#### Perceived hand temperature change.

There was a significant main effect of stimulation intensity on perceived hand temperature change (χ^2^ = 1,635.32, *P* < 0.001). Post hoc analyses revealed that the perceived temperature change was significantly higher for all stimulation conditions (25%, 50%, 75%, and 100%) compared to the 0% stimulation intensity (smallest *b* = −10.57, smallest *t* = −5.19, all *P* values < 0.001). Furthermore, perceived hand temperature change was significantly higher for the 50% compared to the 25% stimulation intensity (*b* = −27.74, *t* = −13.59, *P* < 0.001), for the 75% compared to the 50% stimulation intensity (*b* = −21.99, *t* = −10.77, *P* < 0.001), and for the 100% compared to the 75% stimulation intensity (*b* = −8.36, *t* = −4.101, *P* < 0.001) (Fig. 3*B*).

#### Change in thermal interoceptive accuracy.

There was a significant main effect of stimulation intensity on thermal interoceptive accuracy (χ^2^ = 99.69, *P* < 0.001). Post hoc analyses revealed that participants were more accurate at baseline compared to all other stimulations (smallest *b* = 14.17, smallest *t* = 7.42, all *P* values < 0.001). There was no significant difference in participants’ capacity to detect temperature changes across the other stimulation intensities (all *P* values > 0.05) (Fig. 3*C*).

### Cardiac and Thermal Interoception

#### Characterization of thermal interoception.

Considering that thermal interoceptive accuracy did not vary across active stimulations (25%, 50%, 75%, and 100%), which all differed from the baseline stimulation (0%), we used only the active stimulations to compute a general measure of thermal interoceptive accuracy. Our measure of thermal interoceptive accuracy was generally quite high (mean = 73.07, standard deviation = 16.45), presented high interindividual variability (ranging from 41.44 to 91.59), and was internally consistent across different types of stimulation intensity (α = 0.820). Thermal interoceptive awareness was also high (mean = 84.96, standard deviation = 13.49), ranging from 53.21 to 99.36.

#### Characterization of cardiac interoception.

Cardiac interoceptive accuracy (mean = 47.81, standard deviation = 23.45) was also internally consistent (α = 0.928) and presented high interindividual variability, ranging from 0 to 97.8. Cardiac interoceptive awareness was high (mean = 0.88, standard deviation = 0.96), varying from 33.27 to 97.34.

#### Comparison and covariation between thermal and cardiac interoception.

**Figure 4.**
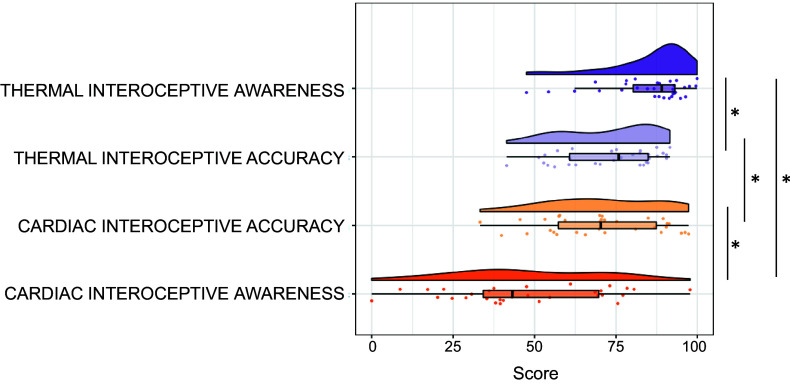
Rain cloud plots displaying the distribution of raw data, box plots, and split violin plots for thermal and cardiac interoception (accuracy and awareness scores). Values closer to 100 indicate higher scores. *Significant difference in scores at *P* < 0.001.

The distribution of cardiac and thermal interoception scores concerning accuracy and meta-awareness is represented in Fig. 4.

Thermal interoceptive accuracy scores were significantly higher than cardiac interoceptive accuracy (*t* = −5.17, *P* < 0.001). Thermal interoceptive awareness scores were also significantly higher than cardiac interoceptive awareness (*t* = −3.85, *P* < 0.001).

Thermal interoceptive accuracy did not correlate with *thermal interoceptive meta-awareness* [*r* = 0.30, *P* = 0.090, Bayes factor (BF) = 0.871], and similarly cardiac interoceptive accuracy did not correlate with cardiac interoceptive awareness (*r* = −0.25, *P* = 0.165, BF = 0.554). For both correlations, Bayesian analysis provided anecdotal evidence (64). With respect to our main analysis, we found that thermal and cardiac interoceptive accuracy did not correlate with each other (*r* = 0.08, *P* = 0.676, BF = 0.239), with BF providing substantial evidence. Finally, thermal interoceptive awareness did not correlate with cardiac interoceptive awareness (*r* = −0.28, *P* = 0.123, BF = 0.685), again with BFs providing only anecdotal evidence.

### The Role of Thermosensitivity and Interoceptive Sensibility in Predicting Thermal Interoceptive Accuracy

#### Thermosensitivity and thermal interoceptive accuracy.

**Figure 5.**
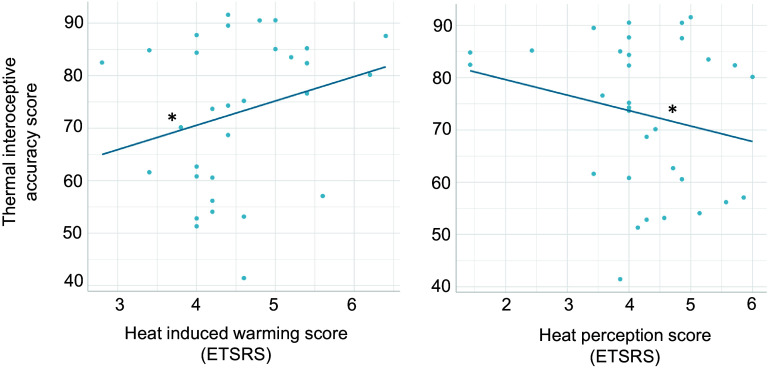
Linear relationship between thermal interoceptive accuracy and self-reported thermosensitivity. Thermal interoceptive accuracy is positively predicted by scores in Heat-Induced Warming (*left*) and negatively predicted by Heat Perception (*right*) in the Experienced Temperature Sensitivity And Regulation Scale (ETSRS). *Significant slope at *P* ≤ 0.01.

The first linear regression analysis investigated whether performance in the thermal interoception task was predicted by subjective thermosensitivity (High Temperature Sensitivity, Solitary Thermoregulation, and Social Thermoregulation of the STRAQ-1 questionnaire; Heat Perception and Heat-Induced Warming of the ETSRS questionnaire). The results show that thermal interoceptive accuracy was predicted by Heat Perception (*b* = −7.448, *t* = −2.315, *P* = 0.009) and Heat-Induced Warming (*b* = 9.51, *t* = 2.79, *P* = 0.010) but not by High Temperature Sensitivity, Solitary Thermoregulation, or Social Thermoregulation (all *P* > 0.05) (Fig. 5).

#### Interoceptive sensibility and thermal interoceptive accuracy.

**Figure 6.**
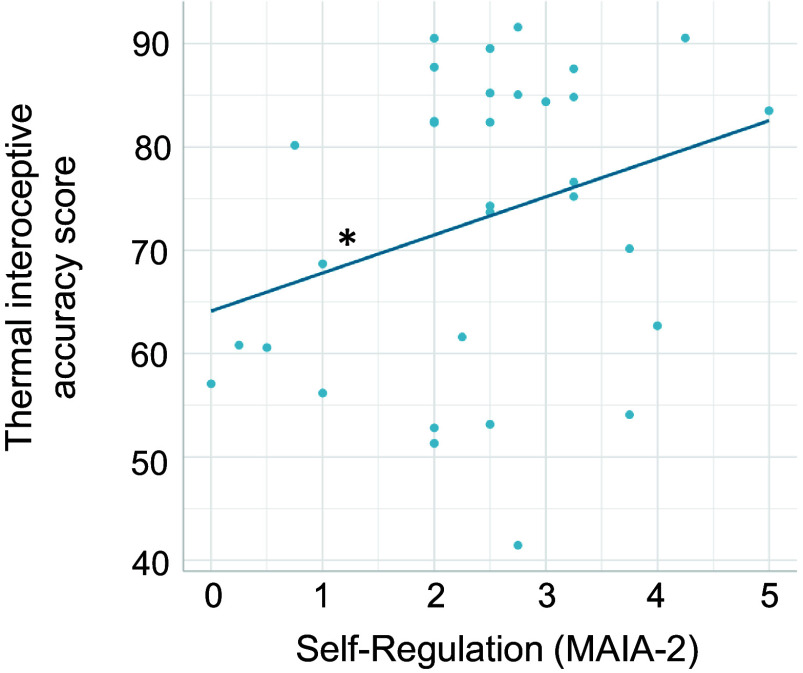
Linear relationship between thermal interoceptive accuracy and self-reported interoceptive sensibility. Self-Regulation [Multidimensional Assessment of Interoceptive Awareness (MAIA-2)] positively predicted thermal interoceptive accuracy. *Significant slope at *P* < 0.05.

The second linear regression analysis investigated whether task performance was predicted by subjective thermosensitivity. The results show that thermal interoceptive accuracy was predicted by Self-Regulation of the MAIA-2 (*b* = 9.07, *t* = 2.41, *P* = 0.024). There was no significant effect of Noticing, Not-Distracting, Not-Worrying, Attention Regulation, Emotional Awareness, Body Listening, Trusting, or the BPQ-SF on thermal interoceptive accuracy (all *P* > 0.0) (Fig. 6).

## DISCUSSION

In the present study we report on the development of a task to measure awareness of changes in peripheral body temperature. In 15 trials, participants received 20-s infrared thermal stimulations to the palm of their right hand. We used five different temperature intensities (0%, 25%, 50%, 75%, and 100% of the infrared light bulb’s maximum intensity), each presented three times. After each stimulation, participants judged how much their hand temperature had changed after the stimulation (perceived hand temperature change). Participants’ responses were compared to a standardized hand temperature change, derived from the real hand temperature change in degrees Celsius as measured with a thermal camera. From this comparison we were able to derive a measure of thermal interoceptive accuracy.

### Measuring Awareness of Changes in Peripheral Body Temperature

The average real hand temperature change was greater with each increasing stimulation intensity, evidencing that our thermal manipulation was effective in producing incremental thermal variations in the participants’ hands. Mirroring these results, participants also reported perceiving increasingly larger temperature changes (perceived hand temperature change) as the intensity of the stimulations increased. For what concerns our measure of thermal interoceptive accuracy, it showed good interindividual variability, which suggests its potentiality as a tool for the assessment of each individual’s thermal awareness. Although participants were generally better at detecting when there was no (or a very low) change in hand temperature after the baseline stimulation (0% stimulation) compared to all other stimulation conditions, there was no significant difference in their ability to accurately detect temperature changes following the other stimulation intensities (i.e., 25%, 50%, 75%, and 100%). This result suggests that thermal interoceptive accuracy is stable across stimulation intensities. Our findings also show that thermal interoceptive accuracy did not correlate with thermal interoceptive awareness. This is in line with evidence supporting the idea that interoceptive accuracy and awareness generally rely on distinct, unrelated processes, with the exception of participants who score very high in interoceptive accuracy (56).

Thermal interoceptive accuracy was positively predicted by participants’ self-reported perception of how quickly or intensely different body parts, e.g., hands, feet, or the torso, become warm when exposed to warm environments compared to other people (Heat-Induced Warming from the ETSRS questionnaire), and negatively predicted by the tendency to always feel warm in different types of environments and/or situations, e.g., indoors, outdoors, in bed, when concentrating, or when watching TV or reading (Heat Perception from the ETSRS), suggesting that the task construct indeed reflects a general, and more accurate, awareness of changes in body temperature. It was not predicted by how bothered by high temperatures participants are (High Temperature Sensitivity from the STRAQ-1), which reflects a more qualitative aspect associated with experiencing warm temperatures, or by Solitary Thermoregulation or Social Thermoregulation (from the STRAQ-1). However, thermal interoceptive accuracy was also positively predicted by the ability to self-regulate through attention to body sensations (Self-Regulation of the MAIA-2), highlighting a potential link between body temperature awareness and self-regulatory behavior. Future research should better investigate the relationship between thermal awareness and the tendency to take action to maintain optimal body temperature and homeostasis. Finally, task performance was not significantly predicted by other MAIA-2 subscales (including subscales of basic body awareness i.e., Noticing or Body Listening) or by the BPQ-SF, suggesting that just like cardiac interoceptive accuracy, thermal interoceptive accuracy is not directly related to general interoceptive sensibility.

### Thermoception and Cardiac Interoception

The present study also shows that thermal interoceptive accuracy (performance in the thermoception task) did not correlate with cardiac interoceptive accuracy (performance in the heartbeat counting task), nor did thermal and cardiac interoceptive awareness. Similarly, Crucianelli and colleagues (21) also observed the absence of a significant association between performance in a thermal matching task and that in the heartbeat counting task. Crucially, the results of these studies align with evidence consistently suggesting that conscious perception of interoceptive signals may differ across sensory channels (18–20). Very importantly, and also considering limitations with traditional heartbeat-associated measures (24), these findings should admonish against using cardiac interoception (or any measure related to a specific sensory domain) as a proxy for interoceptive accuracy in general and should encourage researchers to rely on multiple interoceptive tests when assessing how different individuals process visceral signals or to focus on a limited number of modalities only when driven by specific hypotheses.

We also found that the participants were more accurate in the thermoception task compared to the heartbeat counting task. It is possible that the difference between the two performances reflects the saliency, and possibly a greater awareness, of thermal signals (compared to cardiac signals) in daily life. Indeed, heartbeats produce faint and transient signals that we are often unaware of, as they become salient only under specific circumstances, such as physical exertion, tachycardia, and panic attacks (65). Thus, people are not accustomed to paying attention to their heartbeat as they are required to do during the HCT. Oppositely, the great variability of the environmental temperatures we dwell in requires individuals to take direct, behavioral actions to control body temperature and preserve thermoneutrality. As such, we argue that people may be more accustomed to recognizing temperature fluctuations than they are to recognizing cardiac fluctuations, possibly explaining the difference in performance we observed in our two interoceptive tasks and contributing to the ecological validity of the task as a measure of body awareness.

### Conclusions and Future Directions

Conclusively, we developed a task measuring awareness of changes in peripheral body temperature (of participants’ right hand) through a safe, noninvasive stimulation procedure that did not cause excessive discomfort (based on informal feedback provided by participants) and eliminated tactile confounds by using radiant thermal stimulation. Compared with other measures of awareness of bodily signals that are based on invasive procedures, such as air load judgments (66) or gastric (67, 68) and bladder (69) balloon distension procedures, the noninvasiveness of the present task constitutes an important advantage for its application to the field of neurophysiology and specifically to the interoceptive one. Importantly, the task presented here may prove especially relevant for studying the role of thermal awareness in thermoregulatory behavior (31, 32) and in psychopathologies characterized by altered thermoregulatory capacities [e.g., sleep disturbances (33–35), mood disorders (36–38), eating disorders (39, 40), and schizophrenia (41)]. Furthermore, the task may also be relevant to study of emotionally arousing (42–47) and social situations such as social connection, (48, 70) social exclusion (49, 50, 71, 72), and dishonest behavior (51, 52). However, further research is needed to disentangle whether the processing of changes in peripheral body temperature (as addressed in this task) relies on processes similar to or distinct from those involved in shifts in whole body and facial temperature. These processes may be more relevant in emotional and social contexts, and it will be crucial to investigate whether the origin of temperature changes (endogenous vs. exogenous) affects our awareness. Future studies may explore with dedicated technologies, such as ingestible sensors measuring inner temperature (73, 74), whether peripheral thermoception is associated with interoception of one’s internal core temperature. Furthermore, future research is needed to disentangle whether performance in this task relates to how people perceive changes in facial temperature (e.g., exogenous changes induced by heat lamps on participants’ faces or endogenous changes induced by arousing tasks aimed at increasing face temperature) and in whole body temperature (e.g., by exogenous changes caused by room temperature or endogenous changes caused by exercise or social contact). If the perception of temperature proves to be similar across different body areas (peripheral, facial, and whole body), our task may provide an effective and ecological measurement of general thermosensitivity. Finally, whereas our study investigated the relationship of thermoception with cardiac interoception, more research is needed to further understand the way in which the present task relates to other measures of awareness of the physiological states of the body. Particularly, as temperature perception is often mediated by the skin, it is crucial to assess the relationship of our task with other measures of touch-mediated thermosensitivity (61) (e.g., Ref. 73).

## DATA AVAILABILITY

The anonymized datasets analyzed in the present study are available in the OSF repository, https://osf.io/6bzm4/?view_only=fbe54a3e1e4d4259bc4849fc14db1c00. The scripts used for presenting the experimental stimuli and collecting participants’ responses as well as for analysis are available upon request to the corresponding author (alishavabba@gmail.com).

## GRANTS

The study was supported by European Research Council (ERC) Advanced Grant (eHONESTY, Prot. 789058) to S.M.A.

## DISCLOSURES

No conflicts of interest, financial or otherwise, are declared by the authors.

## AUTHOR CONTRIBUTIONS

A.V., M.S.P., M. Scattolin, G.P., and S.M.A. conceived and designed research; A.V., M. Scattolin, and M. Spitaleri performed experiments; A.V. and M. Spitaleri analyzed data; A.V., M.S.P., M. Scattolin, and G.P. interpreted results of experiments; A.V. prepared figures; A.V. drafted manuscript; M.S.P., M. Scattolin, G.P., and S.A. edited and revised manuscript; A.V., M.S.P., M. Scattolin, M. Spitaleri, G.P., and S.M.A. approved final version of manuscript.
